# Effects of body mass index on IVF outcomes in different age groups

**DOI:** 10.1186/s12905-023-02540-8

**Published:** 2023-08-09

**Authors:** Dan Liu, Li Li, Ningyu Sun, Xiaole Zhang, Ping Yin, Wuwen Zhang, Panwei Hu, Hua Yan, Qinhua Zhang

**Affiliations:** 1 Department of Reproductive Medicine, Shuguang Hospital, affiliated to Shanghai University of Traditional Chinese Medicine, Shanghai, China; 2 Department of Gynecology, Shuguang Hospital, affiliated to Shanghai University of Traditional Chinese Medicine, Shanghai, China

**Keywords:** BMI, Age, IVF, Pregnancy outcomes

## Abstract

**Background:**

Herein, we aimed to analyse the effects of body mass index (BMI) on the treatment outcomes of in vitro fertilisation (IVF) in a cohort of women undergoing their first IVF cycle.

**Methods:**

A total of 2311 cycles from 986 women undergoing their first IVF/intracytoplasmic sperm injection cycle with fresh/frozen embryo transfer between January 2018 and December 2021 at the Center of Reproductive Medicine, Shuguang Hospital affiliated to Shanghai University of Traditional Chinese Medicine, were considered in this retrospective cohort study. First, the included patients were classified into four groups based on their BMI: underweight (BMI < 18.5 kg/m^2^, 78 patients), normal weight (18.5 ≤ BMI < 24 kg/m^2^, 721patients), overweight (24 ≤ BMI < 28 kg/m^2^, 147 patients), and obese (BMI ≥ 28 kg/m^2^, 40 patients). The IVF outcomes included the Gn medication days; Gn dosage; number of retrieved oocytes, mature oocytes, fertilized oocytes, cleavages, and available embryos and high-quality embryos; implantation rate; clinical pregnancy rate and live birth rate. Next, all the obtained data were segregated into three different subgroups according to the patient age: < 30 years, 30–38 years and > 38 years; the IVF pregnancy outcomes were compared among the groups.

**Results:**

Compared with the other three groups, the underweight group had a higher number of fertilized oocytes, cleavage and available embryos and a smaller Gn medication days and required a lower Gn dosage. There was no difference in the number of retrieved oocytes and mature oocytes among the groups. Moreover, compared with the women aged 30–38 years in the overweight group, those in the normal weight group had a significantly higher implantation rate, clinical pregnancy rate and live birth rate (*p* = 0.013 OR 1.75, *p* = 0.033 OR 1.735, *p* = 0.020 OR 1.252 respectively). The clinical pregnancy rate was also significantly higher in those aged 30–38 years in the normal weight group than in the obese group (*p* = 0.036 OR 4.236).

**Conclusions:**

Although the BMI can greatly affect the pregnancy outcomes of women aged 30–38 years, it has almost no effects on the outcomes of younger or older women.

## Background

Obesity is a chronic disease with an increasing prevalence worldwide; it also negatively affects female fertility [1]. Compared with normal weight women, overweight or obese women are at a higher risk of infertility, abortion, pre-eclampsia, gestational diabetes and other pregnancy-related or obstetric complications [2, 3]. Obese women are more likely to experience ovulatory dysfunction, disruptions in the hypothalamic–pituitary–ovarian axis, and oocyte quality defects [4]. Increasing evidence has shown that obesity affects clinical outcomes after in vitro fertilization (IVF) procedures [5].

Obesity is usually determined based on the body mass index (BMI), calculated as weight in kilograms divided by height in metres squared [6]. According to the World Health Organization, normal weight is defined as 18.5 ≤ BMI ≤ 24.99 kg/m^2^, overweight as 25 ≤ BMI ≤ 29.9 kg/m^2^, and obesity as BMI ≥ 30 kg/m^2^ [7]. However, according to the Chinese Ministry of Health, the Chinese have a lower BMI than comparable European populations; thus, BMI as an indicator of modern metabolic diseases functions different in the Chinese. The Working Group on Obesity in China has formulated its own BMI classification criteria, defining underweight as BMI < 18.5 kg/m^2^, normal weight as 18.5 ≤ BMI < 24 kg/m^2^, overweight as 24 ≤ BMI < 28 kg/m^2^, and obesity as BMI ≥ 28 kg/m^2^ [8].

Age is recognised as an independent negative prognostic factor for IVF outcomes [9, 10]. Therefore, we aimed to evaluate the effects of BMI on IVF pregnancy outcomes based on the patients’ age group.

## Materials and methods

### Study design and participants

This retrospective study was conducted at the Center of Reproductive Medicine, Shuguang Hospital affiliated to Shanghai University of Traditional Chinese Medicine, Shanghai, China. A total of 2311 cycles from 986 women undergoing their first IVF/intracytoplasmic sperm injection (ICSI) cycle with fresh/frozen embryo transfer between January 2018 and December 2021 were included. Blastocyst transfers, cycles using pre-implantation genetic testing aneuploidy and patients with polycystic ovarian syndrome were excluded. The study was approved by the Institutional Research and Ethics Board of the Shuguang Hospital affiliated to Shanghai University of Traditional Chinese Medicine (Study 12–283-SDR). Cycle information was obtained from the Shuguang reproductive centre database. The patients’ IVF/ICSI pregnancy outcomes were obtained from their medical records or through telephonic conversations. The cycle characteristics and IVF/ICSI outcomes (the number of retrieved oocytes, mature oocytes, fertilized oocytes, cleavages, available embryos and high-quality embryos; implantation rate; clinical pregnancy rate and live birth rate) were compared among patients with different BMIs and of different ages.

All women underwent one of the following four ovarian hyperstimulation protocols: (1) A microdose flare-up gonadotropin-releasing hormone (GnRH) protocol on days 2–3 of the cycle with gonadotropin(Gn)(Merck, Germany) stimulation on the third day of GnRH agonist(Triptorelin acetate injection,Ferring, Switzerland) administration; (2) a flexible, antagonist protocol with Gn stimulation beginning on days 2–3 of the cycle with a GnRH antagonist(Cetrorelix acetate powder for injection, Merck, Germany) administered when at least one or more follicles grew to 14 mm; (3) a mid-luteal, long agonist protocol with a GnRH agonist beginning in the mid-luteal phase and Gn stimulation after 2 weeks of downregulation; (4) a mild protocol using clomiphene citrate on days 2–3 of the cycle with Gn stimulation. Human chorionic gonadotropin (hCG) (Merck, Germany) was administered to stimulate the final stages of follicular development when the follicles reached maturity, i.e., when the leading follicle grew to > 18 mm. Transvaginal follicle aspiration was performed 36 h after the hCG administration. Conventional insemination or ICSI was performed using standard techniques.

The embryos were then cultured for 3 days until the cleavage stage was reached. Fertilisation was assessed on day 3, and the oocytes were enumerated as either normally fertilised (two pronuclei) or abnormal fertilised (one or three pronuclei). Next, the cleavage-stage embryos were scored based on the cell number and fragmentation degree, according to Hardarson’s grading system (2001) [11]. At an appropriate developmental stage, with < 20% fragments and a mild degree of uneven-sized blastomeres (grade I, IIA and IIB), the embryos reached the day-3 embryo stage, indicating that they were suitable for transfer. For fresh embryo transfer cycles, luteal support was initiated after oocyte extraction, and the embryos were subsequently transferred into the uterus. For frozen embryo transfer cycles, luteal support started on the day of endometrial transformation. Progesterone sustained-release vaginal gel (Merck, Germany) (90 mg/d) was used for 2 weeks to provide luteal support. The serum β-hCG levels were assessed 14 days after the embryo transfer.

### Data set

The BMI was measured prior to hormonal stimulation. The patients were divided into four groups according to their BMI and accordingly administered treatment cycles: underweight (BMI < 18.5 kg/m^2^, 78cycles), normal weight (18.5 ≤ BMI < 24 kg/m^2^, 721 cycles), overweight (24 ≤ BMI < 28 kg/m^2^, 147 cycles) and obese (BMI ≥ 28 kg/m^2^, 40 cycles). All the obtained data were categorised into three groups based on the patient age: < 30 years, 30–38 years and > 38 years.

The following patient information was collected: age; type of infertility; primary diagnosis; the total number of IVF/ICSI cycles, fresh/frozen embryo transfer cycles, stimulation days, oocytes retrieved, mature oocytes, fertilized embryos, cleavages and available embryos and high-quality embryos and the total Gn dosage.

### Outcome measures

The primary outcome was the clinical pregnancy rate, defined as the presence of an intrauterine gestational sac with an embryonic pole with a foetal heartbeat on transvaginal ultrasound. The secondary outcomes included the implantation rate (number of ultrasound-identifiable sacs divided by the number of embryo transfers), live birth rates (delivery of a live-born infant at ≥ 24 weeks of gestation), Gn dose and the number of oocytes and embryos. Implantation was confirmed by a positive serum pregnancy test (serum β-hCG). Ectopic pregnancies were confirmed through the ultrasonographic presence of an intrauterine sac at 6–8 weeks of gestation. Spontaneous abortion was defined as the loss of a clinical or ongoing pregnancy before 20 weeks of gestation.

### Statistical analysis

SPSS 26.0 software was used to analyse the data. Prior to statistical comparisons, normality tests were used to determine whether the data were normally distributed. Normally distributed measurement data are indicated as mean ± standard deviation ($\overline{x }$ ± S). Inter group comparisons were analysis by using variance (ANOVA). The Kruskal–Wallis test was used for independent between-group comparisons, and the constituent ratio or rate (%) was calculated for the enumeration data. The non-parametric, chi-square, and Cochran–Mantel–Haenszel tests were used for comparing the continuous and categorical variables between groups. *P* < 0.05 was considered statistically significant.

## Results

### Comparison of the patients’ baseline characteristics

This study included a total of 2311 fresh or frozen embryo transfer cycles from 986 patients, resulting in 325 clinical pregnancies and 277 live births. The patients’ demographics and treatment characteristics were compared across the BMI groups (Table 1). There were no statistically significant differences in the type of infertility, primary diagnosis, total number of IVF/ICSI cycles and total number of fresh/frozen embryo transfer cycles. The age difference among the four groups was statistically significant (*P* < 0.05).

**Table 1:** Comparison of the patients’ baseline characteristics

Variable	BMI (kg/m^2^)				*P* Value
	BMI＜18.5	18.5≤BMI＜24	24≤BMI＜28	BMI≥28	
Number of patients	78	721	147	40	0.004*
Age (mean±SD)	32.56±4.62	34.21±5.12	34.33±4.77	32.30±5.07	
BMI (mean±SD)	17.80±0.63	20.93±1.40	25.29±0.99	30.52±2.68	
Type of infertility: n (%)					
Primary infertility	54 (69.2)	421 (58.4)	80 (54.4)	25 (62.5)	0.176
Secondary infertility	24 (30.8)	300 (41.6)	67 (45.6)	15 (37.5)	
Primary diagnosis: n (%)					
Tubal factors	58 (74.4)	537 (74.5)	105 (71.4)	29 (72.5)	0.457
Male factor	13 (16.7)	100 (13.9)	30 (20.4)	7 (17.5)	
Uterine factor	0 (0)	18 (2.5)	2 (1.4)	1 (2.5)	
Other/unexplained	7 (8.9)	66 (9.1)	10 (6.8)	3 (7.5)	
Total number of fresh IVF/ICSI cycles	93	905	187	56	
Fresh IVF/ICSI cycles (mean±SD)	1.19±0.51	1.26±0.53	1.27±0.54	1.40±0.54	0.592
Total number of frozen embryo transfer	88	785	154	43	
Frozen embryo transfer (mean±SD)	1.35±0.59	1.31±0.61	1.38±0.70	1.39±0.67	0.097

Data are presented as mean±standard deviation or n (%).*P < 0.05 as compared with the control group

### Comparison of the patients’ IVF characteristics

**Fig. 1:**
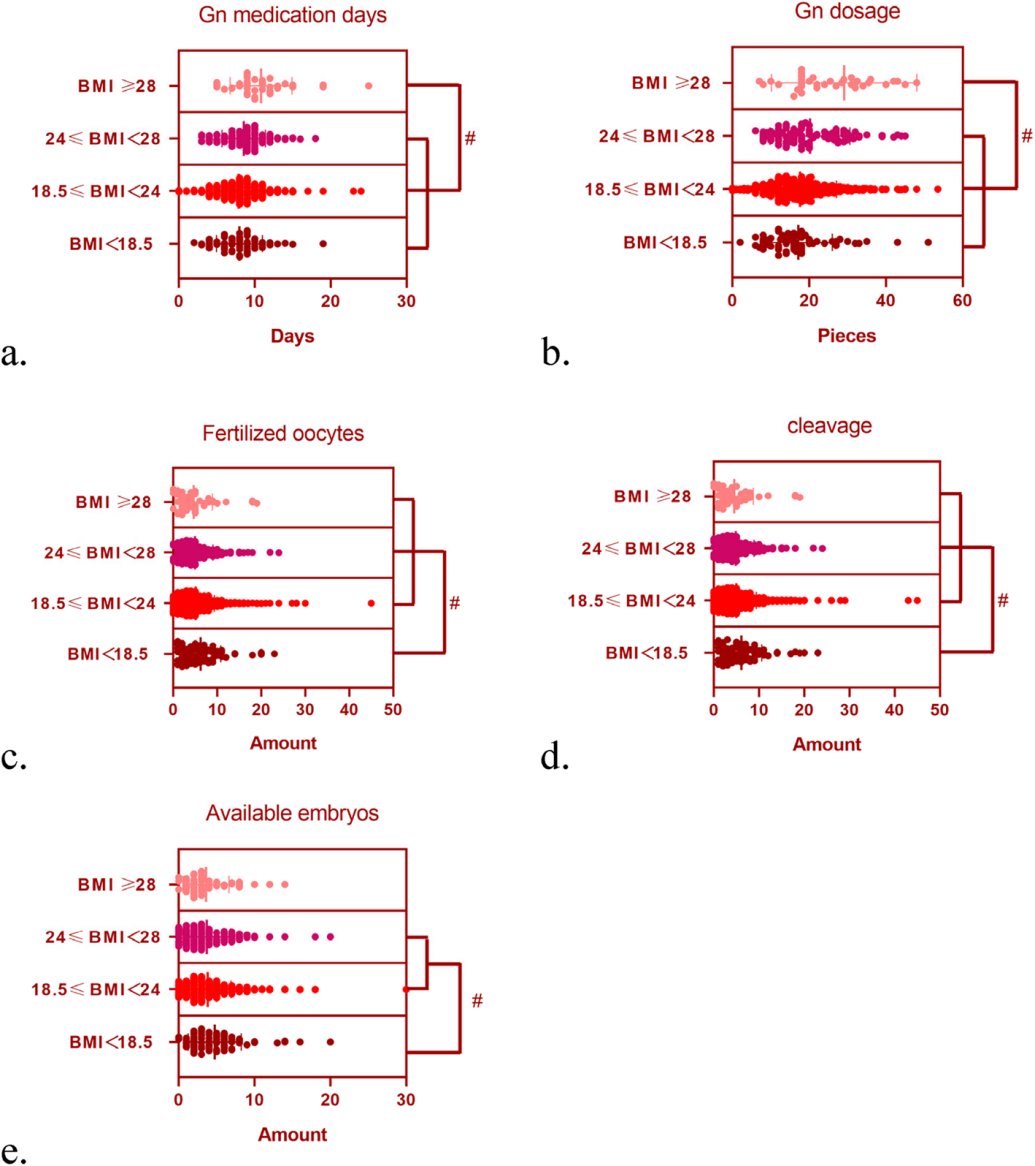
IVF/ICSI cycle characteristics of the patients according to their BMI. Note: Compared with the other groups, the obese group had of the longest Gn cycle (*P* < 0.05) (**a**). Compared with the other groups, the obese group had the highest Gn dosage (*P* < 0.05) (**b**). Moreover, the number of fertilized oocytes was the highest in the underweight group (*P* < 0.05) (**c**). The number of cleavages was the highest in the underweight group (*P* < 0.05) (**d**). In addition, the number of available embryos was statistically higher in the underweight group than in the normal weight and overweight groups (*P* < 0.05) (**e**)

The IVF/ICSI characteristics included the Gn medication days, Gn dosage and number of retrieved oocytes, mature oocytes, fertilized oocytes, cleavages and available embryos and high-quality embryos. There were no statistically significant differences in the number of retrieved oocytes, mature oocytes and high-quality embryos among the underweight, normal weight, overweight, and obese groups (*P* > 0.05). The obese group had the longest Gn medication days among all groups (*P* < 0.05) (Fig. 1a). Compared with the other groups, the obese group had the highest Gn dosage (*P* < 0.05) (Fig. 1b). Moreover, the number of fertilized oocytes was the highest in the underweight group (*P* < 0.05) (Fig. 1c). The number of cleavages also was the highest in the underweight group (*P* < 0.05) (Fig. 1d). In addition, the number of available embryos was statistically higher in the underweight group than in the normal weight and overweight groups (*P* < 0.05) (Fig. 1e) (Table 2).

**Table 2:** IVF/ICSI cycle characteristics of the patients according to their BMI

Variable (mean±SD)	BMI (kg/m^2^)				*P* Value
	BMI＜18.5	18.5≤BMI＜24	24≤BMI＜28	BMI≥28	
Gn medication days	7.98±3.02	7.90±2.84	8.48±2.73	10.83±4.08	0.000*
Gn dosage (piece, 75IU/ piece)	17.16±8.86	17.61±9.52	20.21±10.43	29.11±18.94	0.000*
Retrieved oocytes	7.61±5.39	6.39±5.09	6.34±4.99	5.98±4.98	0.061
Mature oocytes	5.13±3.36	4.91±4.13	4.19±3.07	4.54±2.99	0.576
Fertilized oocytes	6.23±4.60	5.08±4.28	4.94±4.08	4.64±4.27	0.020*
Number of cleavage	6.11±4.49	4.99±4.39	4.83±4.08	4.53±4.21	0.017*
Available embryos	4.76±3.49	3.84±3.08	3.71±3.16	3.60±3.04	0.017*
High quality embryos	3.57±3.14	2.98±2.62	2.89±2.72	2.67±2.62	0.134

Data are presented as mean ± standard deviation. *P < 0.05 as compared with the control group

### Comparison of pregnancy outcomes

**Fig. 2:**
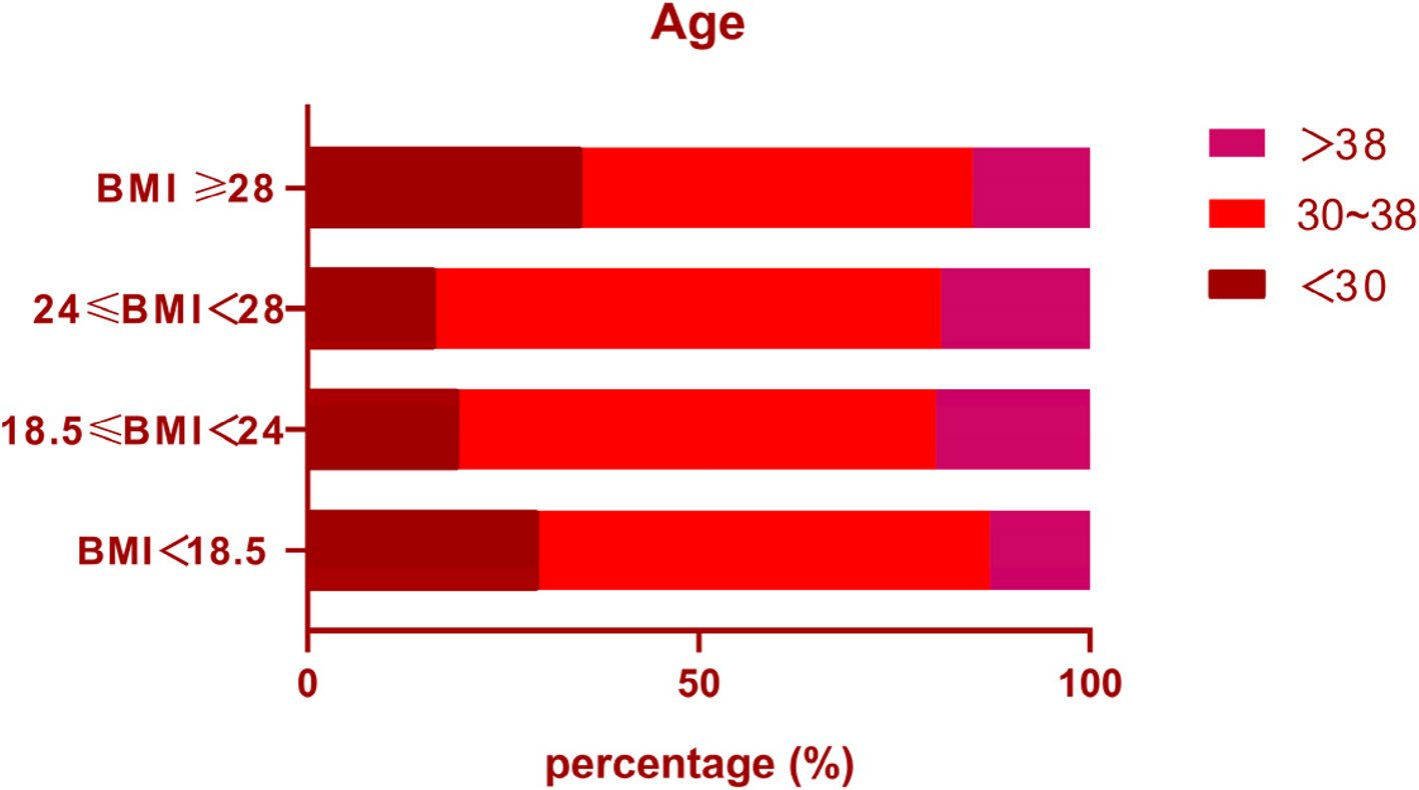
Age distribution of the patients in each group. Note: Most patients were aged 30–38 years and that there were more patients aged < 30 years in the underweight and obese groups

The normal weight group had the highest clinical pregnancy rate (27.3%) and implantation rate (18.1%), and the obese group had a 26.7% spontaneous abortion rate, which contributed to only 60% of the live birth rate in this group (Table 3). This indicates that although obese patients may get pregnant by using assisted reproductive technology,  > 50% of them may be unable to give birth to live children. Underweight patients had a 88.5% live birth rate, which was the highest among the four groups (Table 3). We did age profiles of four groups and found that most of the ages were concentrated in the 30 to 38 years, which may have influenced the results (Fig. 2). It is therefore necessary to stratify by age and further analyse the effect of BMI on pregnancy outcomes.

**Table 3:** Pregnancy outcomes according to BMI

Variable	BMI (kg/m^2^)			
	BMI＜18.5	18.5≤BMI＜24	24≤BMI＜28	BMI≥28
Total Embryos transferred: n (mean±SD)	205 (1.95±0.21)	1712 (1.90±0.30)	339 (1.92±0.28)	96 (1.92±0.27)
Total Sacs	32	310	46	17
Implantation rate (%)	15.6	18.1	13.6	17.7
Ectopic pregnancies	0	2	0	2
Spontaneous abortion: n (%)	0 (0)	32 (13.0)	5 (13.2)	4 (26.7)
Clinical pregnancies: n (%)	26 (24.8)	246 (27.3)	38 (21.5)	15 (30)
Live births: n (%)	23 (88.5)	212 (86.2)	33 (86.9)	9 (60.0)

Data are presented as mean ± standard deviation or n (%)

### Factors affecting the pregnancy outcomes in patients undergoing IVF/ICSI

In order to explore the factors affecting pregnancy outcomes, we conducted a multi-factor analysis: pregnancy outcomes were taken as the dependent variable (success = 1, failure = 0), and factors with significant differences in univariate analysis (age and BMI) were taken as independent variables. Value allocation was performed. Age (age < 30 years = 1, 30 ≤ age ≤ 38 years = 2, age > 38 years = 3) and BMI (BMI < 18.5 = 1, 18.5 ≤ BMI < 24 = 2, 24 ≤ BMI < 28 = 3, BMI ≥ 28 = 4) were included in logistic regression model. Age and BMI were entered into the regression equation, X^2^ = 117.563, *P* < 0.00, so the Logistic regression equation had statistical significance. Both age and BMI were negative factors affecting pregnancy outcome (*P* < 0.05) (Table 4).

**Table 4:** Logistic regression analysis of factors affecting the pregnancy outcomes in patients undergoing IVF/ICSI

Variable	B	SE	Wald	*P*	OR	95%CI
Age	-1.166	0.117	99.252	0.000^*^	0.312	0.248–0.392
BMI	-.230	.115	3.977	0.046^*^	0.794	0.634–0.996

^*^P < *0.05*

### Detailed clinical outcomes classified by age

To exclude the influence of age, the patients were divided into three subgroups: < 30 years, 30 ≤ age ≤ 38 years and > 38 years. The implantation rate, clinical pregnancy rate and live birth rate were significantly higher in women aged 30–38 years in the normal weight group than in the overweight group (*P* = 0.013 OR 1.75, *P* = 0.033 OR 1.735, *P* = 0.020 OR 1.252 respectively) (Table 5). In addition, the clinical pregnancy rate was also significantly higher in the normal weight group than in the obese group in women aged 30–38 years (*P* = 0.036 OR 4.236). In Furthermore, there were no significant differences in the embryo implantation rate, clinical pregnancy rate and live birth rate among other groups (Table 5).

**Table 5:** Detailed clinical outcomes classified by age

Age	Variable	BMI (kg/m^2^)																	
		BMI＜18.5 VS 18.5 ≤BMI＜24			BMI＜18.5 VS 24 ≤BMI＜28			BMI＜18.5 VS BMI≥28			18.5≤BMI＜24 VS 24≤BMI＜28			18.5≤BMI＜24 VS BMI≥28			24≤BMI＜28 VS BMI≥28		
		*χ*^2^	*P*	OR	*χ*^2^	*P*	OR	*χ*^2^	*P*	OR	*χ*^2^	*P*	OR	*χ*^2^	*P*	OR	*χ*^2^	*P*	OR
＜30	Implantation rate	2.261	0.133	1.623	0.025	0.873	1.067	0.131	0.717	1.200	1.892	0.169	0.657	0.497	0.481	0.740	0.057	0.811	0.889
30~38		4.320	0.038	0.547	0.016	0.900	0.956	1.895	0.169	0.528	6.213	0.013	1.750	0.008	0.929	0.967	1.950	0.163	1.810
＞38		0.000	1.000	0.999	0.240	0.636	1.580	1.367	0.512	——	0.538	0.588	1.590	1.409	0.622	——	0.881	1.000	——
*χ*^2^_MH_ (*P* Value)		/			0.000 (0.994)			0.059 (0.808)			/			0.000 (0.992)			0.172 (0.678)		
OR_MH_ (95% CI)		/			1.035 (0.626-1.713)			0.872 (0.452-1.680)			/			0.958 (0.553-1.660)			1.202 (0.642-2.251)		
＜30	Clinical pregnancy rate	0.486	0.486	1.337	0.062	0.804	0.878	0.303	0.582	0.719	1.200	0.273	0.657	1.672	0.196	0.538	0.119	0.730	1.221
30~38		2.100	0.147	0.626	0.044	0.834	1.086	1.562	0.333	2.653	4.566	0.033	1.735	4.393	0.036	4.236	1.382	0.362	0.410
＞38		0.003	1.000	1.045	0.335	0.619	1.744	1.449	0.500	——	0.632	0.427	1.669	1.459	0.610	——	0.866	1.000	——
*χ*^2^_MH_ (P Value)		/			0.000 (0.994)			0.059 (0.808)			/			0.000 (0.992)			0.172 (0.678)		
OR_MH_ (95% CI)		/			1.035 (0.626-1.713)			0.872 (0.452-1.680)			/			0.958 (0.553-1.660)			1.202 (0.642-2.251)		
＜30	Live birth rate	1.814	0.228	0.393	6.830	0.019	——	0.020	1.000	0.875	3.430	0.107	——	1.089	0.373	2.227	6.061	0.037	——
30~38		1.452	0.616	——	4.828	0.061	——	16.000	0.008	——	6.658	0.020	1.252	17.20	0.011	——	4.107	0.111	——
＞38		1.658	0.486	——	0.833	1.000	——	——	——	——	0.206	1.000	1.800	——	——	——	——	——	——
*χ*^2^_MH_ (P Value)		0.003 (0.957)			/			/			/			/			/		
OR_MH_ (95% CI)		1.147 (0.360-3.654)			/			/			/			/			/		

## Discussion

Obesity is closely associated with female infertility and not only leads to difficulties in pregnancy but also increases the risk of abortion and pregnancy-related complications [12]. Because the incidence of obesity is continually rising, more and more overweight and obese women are seeking IVF treatment. Therefore, it is important to explore the effects of different BMIs on the patients’ IVF outcomes and pregnancy outcomes.

Many previous studies have studied the effect of BMI on IVF/ICSI outcomes, but have reached different conclusions, some suggesting that BMI has no effect on pregnancy rate and live birth rate [13, 14], while others suggesting that BMI can lead to a decrease in patients' live birth rate [5]. Compared with previous studies on the relationship between BMI and IVF pregnancy outcomes, the strength of the present study is derived from an analysis that includes age, which is the most important factor determining the oocyte quality and IVF outcomes [10]. Filipa et al. also studied the effect of age and BMI on ART outcomes, and the conclusions they reached were consistent with our findings [15]. The effects of obesity on pregnancy outcomes have been extensively documented [5, 16, 17]. Our results show that both age and BMI were negative factors affecting pregnancy outcome.

The number of retrieved oocytes, fertilized oocytes, and cleavage oocytes in underweight group were higher than those in the other three groups, and the live birth rate was slightly higher than other groups, which may be related to the patient's age. As shown in Fig. 2, the proportion of people under 30 years old n underweight group is higher than that of the normal weight group and overweight group. Therefore, it is necessary to compare the pregnancy outcomes of patients stratified by age.

Further research has found that BMI can greatly affect pregnancy outcomes of women aged 30–38 years undergoing IVF/ICSI, but has almost no influence in the outcomes of younger or older women. The IVF/ICSI clinical pregnancy rate is 1.735 and 4.236 times higher among normal weight patients than among overweight patients and obese patients aged 30–38 years, respectively. However, in women over 38 years old, the negative impact of BMI can be ignored. Therefore, BMI had a negative impact on the pregnancy outcomes of the women who underwent IVF/ICSI in this study, especially those aged 30–38 years. Increased patient age is the most important factor when evaluating the reasons for infertility [18]. Fertility declines starting 35 years of age, and age significantly affects the pregnancy outcomes of women who want to pregnant using assisted reproductive technologies. According to the study results, the negative effects of age > 38 years on pregnancy outcomes may be far greater than those caused by increased BMI. Therefore, even age stratification cannot provide a positive conclusion of the negative effects of BMI. Moreover, the negative effects of BMI are not significant in women aged < 30 years, which may be affected by too many confounding factors. Therefore, it is not comprehensive to exclude only the age factor.

Our study has some limitations. First, the sample size of the obesity group was relatively small, which might have caused deviations in the results. Second, the study only focused on the mothers’ BMIs and ignored the influence of the fathers’ BMIs; thus, the analysis may not have been comprehensive enough. Male obesity, although not significantly, increases the chances of subfertility and leads to lower pregnancy rates [19–21]. Third, ovulation disorders were not detailed in the analysis of infertility factors, which may lead to certain defects when guiding clinical individualised treatment. Fourth, this study included four stimulation protocols, which may have an impact on IVF outcomes. Finally, this study was retrospective, and there was no prior intervention of the investigator, so it was inevitable that there will be bias in the results.

## Conclusions

In conclusion, BMI greatly affects the pregnancy outcomes of women undergoing IVF/ICSI. BMI assessment and appropriate intervention (e.g. diet regulation, physical exercise and effective medically assisted treatments such as acupuncture, traditional Chinese medicine and surgery) before IVF/ICSI treatment, especially among women aged 30–38 years, can positively affect the pregnancy outcomes [22, 23]. Although the pregnancy outcomes of underweight women were not worse than those of normal weight women, BMI was still seen to be a potential influencing factor.

## Data Availability

All data are contained in the manuscript.

## Abbreviations

BMI: Body mass index
IVF: In vitro fertilization
ICSI: Intracytoplasmic sperm injection
Gn: Gonadotropin
GnRH: Gonadotropin-releasing hormone
hCG: Human chorionic gonadotropin
